# Robotic-assisted pyeloplasty in children: a systematic review of the literature

**DOI:** 10.1007/s11701-023-01559-1

**Published:** 2023-03-13

**Authors:** Ciro Esposito, Mariapina Cerulo, Benedetta Lepore, Vincenzo Coppola, Daniela D’Auria, Giorgia Esposito, Roberto Carulli, Fulvia Del Conte, Maria Escolino

**Affiliations:** 1 Department of Translational Medical Sciences (DISMET), Pediatric Surgery Unit, “Federico II” University of Naples School of Medicine, Via S. Pansini 5, 80131 Naples, Italy; 2 Faculty of Computer Science, Free University of Bolzano, Bolzano, Italy; 3 Internal Medicine Unit, University of Naples “Federico II”, Naples, Italy

**Keywords:** Pediatric urology, UPJO, Robotic pyeloplasty

## Abstract

Robotic pyeloplasty has become a natural progression from the development of open, then laparoscopic procedures to treat pediatric patients with ureteropelvic junction obstruction (UPJO). Robotic-assisted pyeloplasty (RALP) is now considered a new gold standard in pediatric MIS. A systematic review of the literature retrieved from PubMed and published in the last 10 years (2012–2022) was performed. This review underlines that in all children except the smallest infants, where the open procedure has benefits in terms of duration of general anesthetic and there are limitations in the size of instruments, robotic pyeloplasty is becoming the preferred procedure to perform in patients with UPJO. Results for the robotic approach are extremely promising, with shorter operative times than laparoscopy and equal success rates, length of stay and complications. In case of redo pyeloplasty, RALP is easier to perform than other open or MIS procedures. By 2009, robotic surgery became the most used modality to treat all UPJO and continues to grow in popularity. Robot-assisted laparoscopic pyeloplasty in children is safe and effective with excellent outcomes, even in redo pyeloplasty or challenging anatomical cases. Moreover, robotic approach shortens the learning curve for junior surgeons, who can readily achieve levels of expertise comparable to senior practitioners. However, there are still concerns regarding the cost associated with this procedure. Further high-quality prospective observational studies and clinical trials, as well as new technologies specific for the pediatric population, are advisable for RALP to reach the level of gold standard.

## Introduction

Intrinsic or extrinsic compression of the ureteropelvic junction (UPJ) caused, respectively, by fibrosis/stenosis of the proximal ureter or aberrant lower pole vessels is a common issue in pediatric urology. In the last years, prenatal hydronephrosis has been found in up to 4.5% screening ultrasounds, with UPJO being the cause in up to 41% of cases [1]. Despite a large proportion of UPJO-like (isolated hydronephrosis, with or without dilated calyces) cases being benign in nature and spontaneously resolve [1], approximately 25% of prenatally diagnosed UPJO require surgery [2]. This diagnostic change has significantly decreased the age of pyeloplasty. Many attempts have been made to simplify the procedure and minimize complications since its first description by Anderson and Hynes in 1949 [3]. Until now, the gold standard for the treatment of UPJ obstruction is still the Anderson–Hynes dismembered pyeloplasty, traditionally performed with an open flank approach, which has an overall success rate ranging from 90 to 100% [3, 4]. In 1995, the first reported pediatric laparoscopic pyeloplasty (LP) was performed [4]. Some years later, the technique was confirmed as a safe and effective minimally invasive treatment alternative for UPJO [4], but a challenging procedure in terms of intracorporeal suturing and knotting, ergonomics and learning curve [4, 5]. The continued interest in minimally invasive treatment for UPJO has inspired new questions about the optimal approach for treating this disease. However, the general application of LP in children was very limited, until the da Vinci system became available in 2002 and the first robot-assisted laparoscopic pyeloplasty (RALP) series was evaluated [5]. The last 10 years have been characterized by continuous improvements in robotic surgery for the pediatric population and RALP has become the most performed robotic procedure in pediatric urology [6], with reported success rates of 95%–100% [7, 8]. This review aims to discuss important and somewhat controversial aspects of RALP in children, mainly: surgical indications, success rate and complications, redo surgery, challenging cases, cost considerations, and training and learning curve, along with a special highlight on the future of robotic surgery in the treatment of pediatric ureteropelvic junction obstruction in children.

## Material and methods

This study aimed to review the international literature of the past 10 years, 2012–2022, focused on robotic-assisted pyeloplasty in patients with UPJO. Published material was identified utilizing the PubMed® (National Center for Biotechnology Information, United States National Library of Medicine, National Institutes of Health) database using multiple combinations of the keywords: ureteropelvic junction obstruction in children, pediatric robot-assisted laparoscopic pyeloplasty, pediatric robotic pyeloplasty, pediatric robot-assisted dismembered pyeloplasty, robotic surgery cost in children, cost analysis, learning curve of robotic surgery in children, robotic simulation, weight and age in pediatric robotic pyeloplasty, future of robotics in children. The articles were then read for relevancy and the bibliographies reviewed for additional citations. Article inclusion within this review was determined by the authors after their evaluation, analysis, and interpretation.

## Discussion

### Ureteropelvic junction obstruction (UPJO)

Development of prenatal screening ultrasonography (US) and postnatal imaging have increased the diagnosis of hydronephrosis and, consequently, decreased the age of pyeloplasty. Prenatal hydronephrosis has been found in up to 4.5% screening ultrasounds, while UPJO occurs in 1 per 1000–2000 newborns [9]. UPJO has a male predominance and affects the left kidney in up to 67% of cases, while up to 10% of cases are bilateral [9]. An ultrasound (US) of the urinary tract allows to grade the severity of hydronephrosis and to determine pelvocalyceal dilation and/or renal cortical thinning at birth. In 1993, the Society for Fetal Urology (SFU) suggested a standard US grading system for the evaluation of hydronephrosis, according to urinary tract dilatation and parenchymal thickness [9]. In 2014, the urinary tract dilation (UTD) classification system was introduced, and six parameters started to be evaluated: anterior–posterior renal pelvic diameter, urinary tract dilatation, parenchymal thickness, parenchymal appearance, ureteral status, and bladder status [10]. It should be emphasized that the first postnatal US should be done more than 48 h after birth to ensure it does not underestimate dilation [1]. In addition to US, 99mTc-MAG3 is recommended for neonatal renography and for visualization of kidneys in patients with compromised renal function [10]. However, being functionally immature, neonatal kidneys may show increased residual cortical activity with the simultaneous injection of the radiopharmaceutical and furosemide (F0 study), retaining up to 50% or more of the peak. Such a phenomenon disappears after the age of 3 months.

### Indications

The indications of RALP do not differ from those of open or laparoscopic surgery and include symptomatic obstruction, urinary tract infections, presence of an obstructive pattern on functional renal scan, and/or worsening differential renal function (DRF) [10]. To date, de Waard et al. in 2018 speculated that hypertension should be considered an indication for surgery as the relief of the obstruction cures hypertension in most children with UPJO [11]. A recent study showed that preoperative characteristics, including sex distribution, laterality, preoperative renal pelvis antero-posterior (AP) diameter, and the split functions of the affected kidneys are comparable between LP and RALP [10].

Real concerns, however, have been raised on performing the procedure in very small spaces [12, 13] and its relative value in small children, as well as the influence of body weight on the outcome in children treated with robotic pyeloplasty, since at present, no specific robotic devices have been developed exclusively for pediatric surgery. Although the consensus goes toward a weight cutoff at 10–15 kg, some authors have reported the successful use of robotic technology in patients under 1 year of age and in infants [13–15]. Another topic supporting the limitation of RALP in small children consists of trocar placement due to the restricted space that often causes conflicts between arms. A recent paper has proved the efficacy of 5 mm rather than 8 mm instruments to optimize the available working space [16–18]. However, since monopolar curved scissors are not available for use with 5 mm ports, monopolar hook diathermy is the alternative. Notwithstanding the weight, correct bed positioning, a reproducible trocar placement method (‘kite-like’ configuration), and the use of a ‘tent effect’ seem sufficient to achieve an adequate working space and avoid prolonged operative setup and operative times [13]. Data on younger and lighter infants are encouraging despite all the studies being mostly retrospective.

### Surgical technique and operative time

The preferred approach to UPJO is the dismembered pyeloplasty described by Anderson–Hynes. While the most used approach is the transperitoneal [19–22], some authors advocate the use of a retroperitoneal approach [23, 24]. A recent multicenter prospective study [25] comparing transperitoneal versus retroperitoneal RALP showed that both approaches are safe and effective, with a shorter hospital stay, but with longer operative time in case of crossing vessels for retroperitoneal RALP. However, a univocal consensus on the approach is yet to be defined and remains a matter of personal preference. While a 3–5 cm distance between ports is ideal, in infants this distance is not always possible, so the trocars are placed as far apart as possible. In transabdominal RALP, ports are positioned as follows: one infraumbilical robotic camera port; two 8 mm working ports along the midclavicular line, one in the epigastric region, 2 cm under the subcostal arch, the other in the right/left iliac fossa, above the inguinal ligament; one 5 mm assistant port between the optic port and the lower working port (left pyeloplasties) or between the optics and the upper working port to lift the liver (right pyeloplasties) [25, 26]. For retroperitoneal RALP, ports positioning has been standardized as well [23, 24]. Recently, robot-assisted laparoscopic single-port pyeloplasty has shown to be feasible in noninfant pediatric patients [27, 28]. Of note, new hidden incision endoscopic surgery (HIdES) technique aims to eliminate visible scarring, placing the robotic working port and camera port below the line of a Pfannenstiel incision, while a second working port is placed infraumbilically [29]. A recent comparative cross-sectional study [30] using da Vinci Xi Surgical System® compared the efficacy, safety, and cosmetic outcomes of three-port RALP with the conventional four-port RALP method, showing that the first can be applied with similar success and safety to the latter in all patients, including infants. Three-port RALP involves the use of a percutaneous hitch stitch to hold the renal pelvis and a 14-G angiocatheter via the percutaneous route to place the double-J stent. Concerning the operative time (OT), the experience acquired through the years has shortened the overall OT to the extent that, from 2019, most studies have always reported an overall OT of less than 120 min [7, 12, 13, 31, 32]. Currently under debate is the necessity for urinary diversion with trans-anastomotic ureteral stenting during pyeloplasty. Stenting is mostly performed through double-J stent placed in an antegrade fashion, or cutaneous pyeloureteral (CPU) stents designed to provide effective urinary drainage, prevent secondary anesthesia, prevent bleeding risks, and be cost-effective [33]. However, since RALP has been extended to smaller children and infants, which have shown a higher risk of stent-related complications (stent migration and fragmentation, infection, fever, pain) and discomfort, surgeons have started to perform robotic stentless pyeloplasty [34–36], showing excellent success rates and minimal complications compared to conventional methods, but studies on larger cohorts and with longer follow-up are needed.

### Outcomes

Pediatric RALP success rate ranges from 90 to 100% [20, 22, 24, 32, 36–47]. In comparison to open or laparoscopic pyeloplasty, RALP presents a shorter hospital stay and less use of medication for pain management following the procedure. What was a negative variable before, namely the longer operative times compared to other modalities, is constantly improving above all in centers with high volume and surgeon experience. Early postoperative complications (< 30 days) range from fever, pain, and hematuria (Clavien I) to urinary tract infection, mild or major urinary leaks (Clavien ≥ 2), to stent migration, omental herniation, and ileocecal volvulus (Clavien IIIb) [19, 39, 41, 48]. Late (≥ 30 days) complications were reported in few cases through the literature and were mostly Clavien IIIb (ipsilateral ureteral stenosis, surgical interventions for umbilical hernia, J–J migration, urinary leakage, and stone formation) [48–50].

### Redo RALP

The incidence of persistent or recurrent UPJO after initial pyeloplasty can range from 3 to 11% [51] and there is no consensus regarding the gold standard approach for failed pyeloplasty. Interestingly, literature has shown some qualitative differences between indications for secondary procedures between laparoscopic and robotic pyeloplasty: the first causes more frequently urine leaks, while RALP has complications related to retrograde pyelogram (RPG) and stent placement, possibly because of its technical ease of intracorporeal suturing [52]. In general, the reason for redo pyeloplasty is obstruction due to crossing vessel, intrinsic narrowing, or high insertion and patients often undergo various endoscopic interventions (placement of stents or percutaneous nephrostomy tube (PCN), endopyelotomy, balloon dilation) without resolution [53]. Of note, RALP simplifies the visualization of crossing vessels and their concomitant surgical management. Despite that the management of UJPO recurrence is more challenging and reserved to the most experienced surgeons due to scar tissue formation, fibrosis, and decreased vascularization of the ureter tract, redo RALP is considered an efficient and safe approach with shorter hospital stay and lower complication rate compared to redo open pyeloplasty and a high success rate similar to the outcomes of primary RALP [53–60].

### Challenging cases

Some cases are characterized by complex anatomy and require experienced hands for the dissection of the UPJ without causing vascular lesions. They include complete intrarenal pelvis, high ureteral insertion, or long ureteral stricture, as well as anatomic variations of morphology and position of the kidney as horseshoe kidney (HSK), renal malrotation, ectopic kidney, or duplex kidney with lower moiety UPJO. Esposito et al. [61] gathered the widest series on complex cases in a multicentric study recommending the use of Anderson–Hynes technique with da Vinci Xi, as it can easily adapt to: vascular anatomy of UPJ in patients with HSK and ectopic kidney, presence of crossing vessels, cases with lower pole UPJO in which the vascularity of the upper moiety ureter should be carefully preserved to avoid stenosis, as well scar fibrotic tissues for recurrent UPJO after failed open pyeloplasty. With a special eye on HSK, which has an increased incidence of UPJO for high insertion of the ureter into the renal pelvis, anatomic relation of the ureter with HSK isthmus, multiple aberrant crossing vessels, urolithiasis, and an increased risk of trauma, a recent multicentric study by the same group [62] showed RALP safety, feasibility and good medium-term outcomes, with an average operative time including docking of 143.5 min (range 100–205), no conversions to laparoscopy or open surgery, no intraoperative complications and overall success rate of 92.8%.

In the adult population, robot-assisted laparoscopic surgery (RALS) has been successfully employed for the treatment of renal stones during concomitant treatment of UPJO and for the primary treatment of staghorn stones [63]. Reports on children with concomitant UPJO and large renal stones [64, 65] demonstrate that simultaneous pyelolithotomy and pyeloplasty in a single surgical session is safe and feasible, despite EAU guidelines recommendation that RALP and surgical treatment for stones should be performed in separate procedures [66].

### Cost considerations

RALP is more expensive than an open procedure, although this cost differential decreased with time and institutional experience. In 2016, Bennett et al. [67] found that charges for operative room time and supplies together with anesthesia time dominate the cost difference between RALP and open pyeloplasty, and that efforts to reduce these specific costs should be the focus of future cost-containment measures. A recent work published in 2021 [68] confirmed a similar cost burden of operating theater, instruments, material, and ward convalescence between open and RALP and justified the use of RALP in a low-volume center. The procedure required for double-J stent removal represented an additional cost. However, in this series, the use of magnetic stents, which avoided the need for further anesthetic procedures, limited costs. Basic cost analysis has shown similar cost between robotic and traditional laparoscopic pyeloplasty [69–72]. However, the high cost of training, maintenance, and materials point to a greater cost for RALP as compared to other modalities [73]. Strategies to lower cost and raise the value of care have been proposed and include: increasing robot utilization, lowering OR turnover time, and optimizing preoperative holding time. All in all, direct costs of RALP, excluding amortization, robotic cost, maintenance, and depreciation, should not be considered as an excessive burden compared to other surgical modalities.

### Training and learning curve

In a meta-analysis by Steinberg et al., the authors found that the learning curve for robotic prostatectomy in adults ranged from 13 to 200 cases, with 77 being the average [74]. Also in pediatric robotics, unfortunately, a standardized robotic curriculum or training protocol is still not available. An open surgeon can quickly attain expertise in RALP by working with a proctor and experienced surgical team [75], but the duration of proctoring needed will vary by individual surgeons and therefore critical self-assessment is essential. Skill acquisition in laparoscopy and robotic pyeloplasty is different in the rate of progression toward proficiency: one study demonstrated that at least 18 cases were required to achieve proficiency in LP, while 13 cases were needed for RALP, with further improvement after 37 cases [45, 46]. Sorensen et al. observed that operative times for RALP were initially longer than open, but became equivalent after 15–20 cases, suggesting that this is the approximate length of the initial learning curve for RALP [76]. Recently, Esposito et al. [7] experienced a learning curve plateau after the first 23 consecutive cases. Moreover, it has been shown how junior surgeons can readily achieve comparable levels of expertise compared with senior practitioners for RALP, assuming proper exposure to robotics and an adequate case volume [77]. A key role should be played by robotic simulation, allowing trainees to learn the basic controls of the instrument and practice surgical skills, as it happens in laparoscopy [77].

### Future considerations

The use of robotic technology has grown in pediatric urology and likely will continue to do so in the future to potentially become the gold standard for certain reconstructive cases, especially RALP. To date, efforts have been made to produce 5 mm instruments, which, however, still have a limited selection, require more working space due to typical joint kinematics, and imply the use of a 5 mm lens that removes the advantageous 3-D image and the 5 mm instruments. For this reason, some authors advocate efforts to improve the quality of 5 mm instruments or to develop 3 mm instruments similar to those used in conventional laparoscopy. Moreover, future trend appears to be moving toward less incisions down to a single-port platform and possibly even no incision in the future [78]. Considering innovation and technology development, augmented reality (AR) and augmented intelligence might represent the next steps in robotic surgery [79, 80].

## Conclusions

RALP demonstrated to be safe and effective, also in children with recurrent UPJO, where it allows an easy identification and consequent dissection of the causes of the initial failed reconstruction, including missed crossing vessels, periureteral fibrosis, and ureteral stricture, as well as in complex anatomical cases [60, 61, 64]. Moreover, RALP shortens the learning curve for junior surgeons, who can readily achieve levels of expertise comparable to senior practitioners [76]. The high cost remains a limitation of robotic surgery, which is the reason why robotic instrumentation is shared between various sub-specialties. However, if from one side this contributes to cost limitation, from the other it reduces the learning curve process. In fact, simulation alone is not sufficient to reach proficiency and a comprehensive training program should consist of simulation and mentorship. The main disadvantage of RALP is the size of robotic instruments, which cause difficulties in the treatment of small children and infants. Despite that some surgeons can overcome this drawback by using 5 mm instruments and others by adjusting port positioning, the final choice should be tailored on surgeon’s preference and customized according to the specific patient. Further high-quality prospective observational studies and clinical trials on RALP in small children, the creation of a pediatric robotic curriculum for learning curve, as well as new technologies and instruments specific for the pediatric population are advisable for RALP to reach the level of gold standard.

## Data Availability

The datasets generated during and/or analysed during the current study are available from the corresponding author on reasonable request.
